# Increase in Urgent Care Center Visits for Sexually Transmitted Infections, United States, 2010–2014

**DOI:** 10.3201/eid2302.161707

**Published:** 2017-02

**Authors:** William S. Pearson, Guoyu Tao, Karen Kroeger, Thomas A. Peterman

**Affiliations:** Centers for Disease Control and Prevention, Atlanta, Georgia, USA

**Keywords:** sexually transmitted infections, STIs, chlamydia, gonorrhea, urgent care, infectious diseases, bacteria, United States, urgent care centers

## Abstract

During 2010–2014, urgent care centers saw a ≈2-fold increase in the number of visits for chlamydia and gonorrhea testing and a >3-fold increase in visits by persons with diagnosed sexually transmitted infections. As urgent care becomes more popular, vigilance is required to ensure proper management of these diseases.

Sexually transmitted infections (STIs) are the most commonly reported nationally notifiable diseases in the United States (*1*), and annual medical costs for these diseases are estimated to exceed $16 billion (*2*). Reported rates of gonorrhea, chlamydia, and syphilis all increased from 2014 to 2015, and antimicrobial drug–resistant gonorrhea remains an important concern (*3*). Therefore, proper diagnosis and treatment of these diseases is essential to reduce STI-associated morbidity rates and prevent further drug resistance (*4*).

Urgent care centers have been identified as appropriate sources of care for nonemergency conditions that would otherwise be treated in a more costly emergency department setting (*5*). These centers are proliferating across the country because of public demand for convenient care and the need to contain healthcare costs (*6*). The Urgent Care Association of America estimates that >9,000 of these centers are currently operating in the United States and, on average, each center sees ≈14,000 visits per year (*7*). Additionally, STI clinics are closing across the country because of decreased funding (*8*); therefore, urgent care centers might increasingly be a typical setting for STI diagnosis and treatment.

We found no literature describing the frequency of diagnosis and treatment of STIs in urgent care settings. Therefore, we set out to estimate the number of visits to urgent care centers for the testing and diagnosis of chlamydia and gonorrhea.

For these analyses, we used data from the MarketScan commercially insured medical claims database for 2010, 2012, and 2014 (*9*). We only included claims for visits to urgent care centers and aggregated these claims to provide numbers of visits for each patient. We then searched the claims for Current Procedural Terminology (CPT) codes and codes from the International Classification of Diseases, Ninth Revision, that indicated the testing or diagnosis of chlamydia, gonorrhea, or an “unspecified venereal disease” (Table). We counted visits that involved a test or diagnosis for each of the indicated diseases for each year and stratified these results by percentage of female patients and the average age of the patients. We then used weights supplied in the dataset and calculated weighted numbers of visits. All analyses were conducted by using SAS 9.3 (**SAS** Institute, Cary, NC, USA).

**Table:** Number of urgent care center visits by commercially insured patients during which the patient was tested for gonorrhea or chlamydia or treated for a diagnosed sexually transmitted infection, United States, 2010–2014*

Characteristic	2010	2012	2014
Total visits†			
Unweighted	1,197,720	2,603,234	4,075,379
Weighted‡	47,000,000	99,000,000	155,000,000
% Female	81.5	81.7	81.9
Average age, y	30.7	35.6	36.3
Visits during which a chlamydia test was performed (CPT codes 87110, 87270, 87320, 87490, 87491, 87492, 87810)			
Unweighted	1,293	2,090	3,711
Weighted	51,701	76,746	136,167
% Female	81.8	79.4	76.6
Average age, y	31.0	29.6	30.7
Visits during which a gonorrhea test was performed (CPT codes 87590, 87591, 87592, 87850)			
Unweighted	1,174	1,885	3,665
Weighted	47,747	69,665	134,403
% Female	82.6	80.1	76.5
Average age, y	31.2	29.8	29.9
Visits during which diagnosed chlamydia was treated (ICD-9 codes 079.88, 079.98, 099.41, 099.50–099.56, 099.59)			
Unweighted	133	430	988
Weighted	4,004	12,152	29,291
% Female	59.4	56.7	61.3
Average age, y	29.7	27.0	26.8
Visits during which a diagnosed “unspecified venereal disease” was treated (ICD-9 codes 099.8, 099.9)			
Unweighted	155	406	1,260
Weighted	4,655	11,392	35,550
% Female	55.3	49.6	50.4
Average age, y	30.1	30.1	31.0
Visits during which diagnosed gonorrhea was treated (ICD-9 code 098)			
Unweighted	116	224	522
Weighted	3,000	6,074	13,783
% Female	41.8	53.5	47.0
Average age, y	30.3	29.1	30.2

*CPT, Current Procedural Terminology; ICD-9, International Classification of Diseases, Ninth Revision; STI, sexually transmitted infection. †Includes all patients seeking care at urgent care centers for any reason. ‡Estimates are rounded to the nearest million.

Overall, we estimated a ≈2.5-fold increase during 2010–2014 for all visits to urgent care centers (Technical Appendix). Among these visits, we observed increases in the numbers of visits that involved STI testing or the treatment of patients with diagnosed STIs. During 2010–2014, a ≈1.5-fold increase occurred in visits that involved chlamydia testing and a ≈2-fold increase in visits involving gonorrhea testing. We observed even larger increases in visits that involved diagnosed STIs. During the same period, we observed a ≈6-fold increase in the numbers of visits that involved diagnosed chlamydia, a >3-fold increase in the numbers of visits that involved diagnosed gonorrhea, and a ≈6-fold increase in the numbers of visits that involved an unspecified diagnosed STI. Most visits that involved STI testing were made by female patients; the average age for all patients at these visits was 28.1 years. Most visits by a patient for diagnosed chlamydia were made by female patients; the average age for all patients at these visits was 27.8 years. The number of visits by patients for an unspecified diagnosed STI was nearly evenly split between male and female patients; the average age of all patients at these visit was 30.4 years. The visits for diagnosed gonorrhea were predominantly made by male patients; the average age of all patients at these visits was 29.9 years.

Visits to urgent care centers have increased over time, and our findings demonstrate that visits to urgent care centers for STI care in particular have dramatically increased. Previous work has highlighted differences in the use of antibiotics to treat chlamydia in emergency departments compared with physician offices (*10*) suggesting that differences might also exist in the treatment of STIs in urgent care centers compared with other healthcare settings. Given the increases in STIs, increases in antimicrobial drug resistance, and increases in use of urgent care centers for STI care, further work is needed to determine how STIs are being managed in this venue to ensure quality care.

Technical Appendix Number of urgent care center visits by commercially insured patients during which the patient was tested for gonorrhea or chlamydia or treated for a diagnosed sexually transmitted infection, United States, 2010–2014.
