# Supplementary material for: Increase in Urgent Care Center Visits for Sexually Transmitted Infections, United States, 2010–2014
Source: Emerg Infect Dis. 2017 Feb;23(2):367–9. doi: 10.3201/eid2302.161707 (PMC5324788; doi:10.3201/eid2302.161707)
Supplement: Technical Appendix — Number of urgent care center visits by commercially insured patients during which the patient was tested for gonorrhea or chlamydia or treated for a diagnosed sexually transmitted infection, United States, 2010–2014. [file 16-1707-Techapp-s1.pdf]

Article DOI: http://dx.doi.org/10.3201/eid2302.161707

Increase in Urgent Care Center Visits for Sexually Transmitted Infections, United States, 2010–2014

Technical Appendix

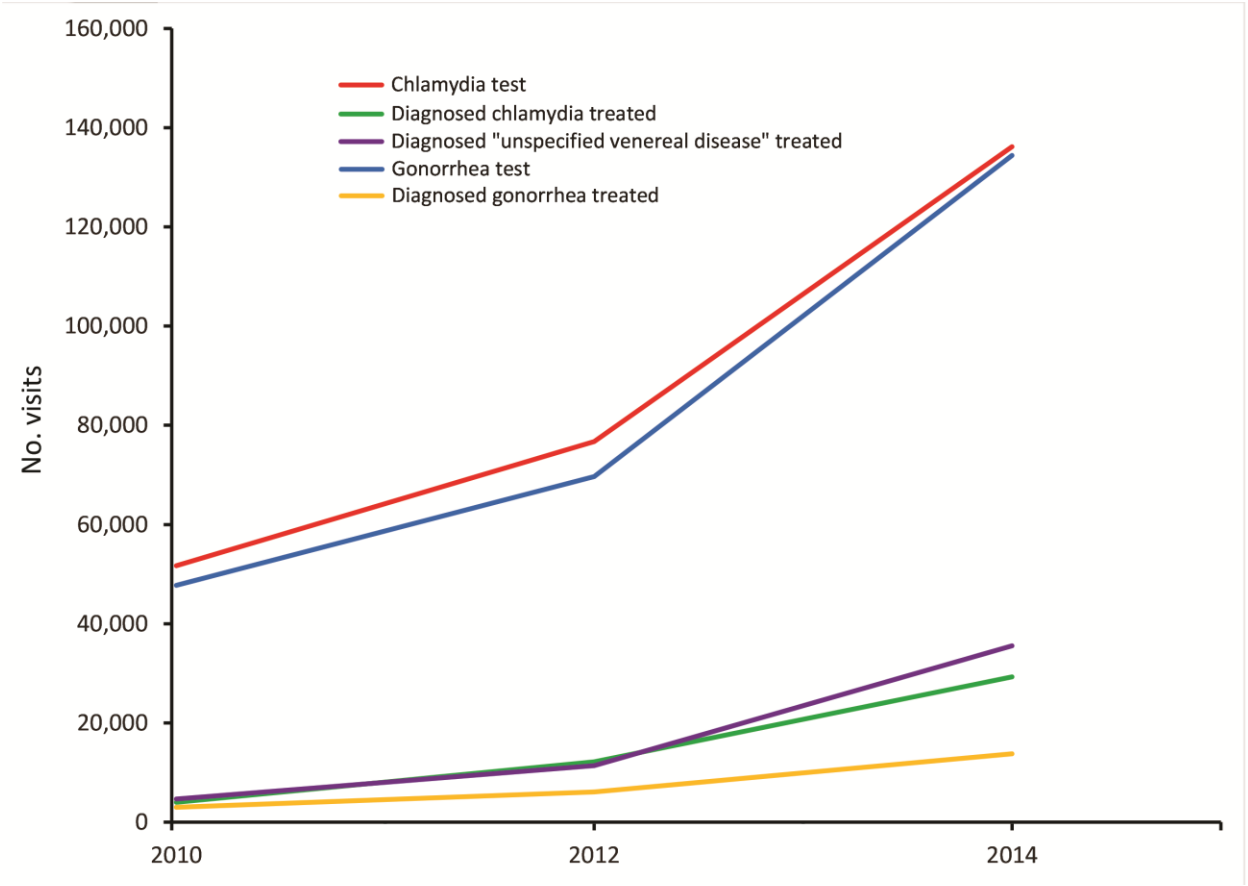

Technical Appendix Figure. Number of urgent care center visits by commercially insured patients during which the patient was tested for gonorrhea or chlamydia or treated for a diagnosed sexually transmitted infection, United States, 2010–2014.
